# Incorporating microglia‐like cells in human induced pluripotent stem cell‐derived retinal organoids

**DOI:** 10.1111/jcmm.17670

**Published:** 2023-01-16

**Authors:** Valeria Chichagova, Maria Georgiou, Madeleine Carter, Birthe Dorgau, Gerrit Hilgen, Joseph Collin, Rachel Queen, Git Chung, Jila Ajeian, Marina Moya‐Molina, Stefan Kustermann, Francois Pognan, Philip Hewitt, Michael Schmitt, Evelyne Sernagor, Lyle Armstrong, Majlinda Lako

**Affiliations:** ^1^ Newcells Biotech Newcastle upon Tyne UK; ^2^ Biosciences Institute Newcastle University Newcastle upon Tyne UK; ^3^ Applied Sciences Northumbria University Newcastle upon Tyne UK; ^4^ F. Hoffmann‐La Roche Ltd Basel Switzerland; ^5^ Novartis Basel Switzerland; ^6^ Merck Healthcare KGaA Darmstadt Germany

**Keywords:** immunocompetent, induced pluripotent stem cell, microglia, retinal organoids

## Abstract

Microglia are the primary resident immune cells in the retina. They regulate neuronal survival and synaptic pruning making them essential for normal development. Following injury, they mediate adaptive responses and under pathological conditions they can trigger neurodegeneration exacerbating the effect of a disease. Retinal organoids derived from human induced pluripotent stem cells (hiPSCs) are increasingly being used for a range of applications, including disease modelling, development of new therapies and in the study of retinogenesis. Despite many similarities to the retinas developed in vivo, they lack some key physiological features, including immune cells. We engineered an hiPSC co‐culture system containing retinal organoids and microglia‐like (iMG) cells and tested their retinal invasion capacity and function. We incorporated iMG into retinal organoids at 13 weeks and tested their effect on function and development at 15 and 22 weeks of differentiation. Our key findings showed that iMG cells were able to respond to endotoxin challenge in monocultures and when co‐cultured with the organoids. We show that retinal organoids developed normally and retained their ability to generate spiking activity in response to light. Thus, this new co‐culture immunocompetent in vitro retinal model provides a platform with greater relevance to the in vivo human retina.

## 1 INTRODUCTION

Microglia are the tissue‐resident macrophages of the central nervous system, including the retina. They arise from a distinct population of primitive myeloid progenitors in the yolk sac prior to the onset of blood circulation.^1^ Histological and more recently transcriptomic analyses of human foetal retina show that microglia migrate into the eye between 6 and 17 weeks of gestation.^2^, ^3^, ^4^ Microglial function in the retina extends beyond the concept of immune defence to their role in development, homoeostasis and disease. In the developing retina, they are involved in shaping neuronal organization, formation and remodelling of synapses and development of vasculature.^5^ This is achieved by maintaining a fine balance between phagocytosis of dying cells and developing neurons, and trophic support to induce cell proliferation and survival.^6^, ^7^, ^8^ In homoeostasis and disease microglia use cytokines as a mode of communication in the microglia‐Muller glia crosstalk, which is essential for mediating adaptive responses following retinal injury.^9^ Disruption to homoeostasis could be associated with ageing and may lead to neurodegeneration.^10^ Changes in microglial cells have also been shown to be a contributing factor to pathophysiological changes associated with several diseases affecting the retina, including diabetic retinopathy,^11^ inherited retinal degeneration^12^, ^13^ and age‐related macular degeneration.^14^

There are currently no adequate human in vitro models that allow the study of the interaction of neural retinal cells and microglia and animal models do not fully recapitulate human retinal physiology. Retinal organoids derived from human induced pluripotent stem cells (hiPSCs) offer a platform to study disease mechanisms and provide a testing tool for drug discovery.^15^, ^16^, ^17^, ^18^, ^19^, ^20^ A potential limitation of the current retinal organoid technology is the lack of the immune cell component, which has been suggested as an underlying factor for the compromised inner retinal lamination.^21^ Since microglia arise from non‐neural lineage, they are unlikely to develop in situ under the existing differentiation conditions. A recent paper has described the development of innate microglia within cerebral organoids; however, their culture conditions included matrigel embedding and reducing the levels of some neuroectodermal stimulants, which encouraged the emergence and maturation of mesodermal progenitors and their differentiation to microglia‐like cells.^22^ Our culture conditions are optimized to direct differentiation of hiPSCs to retinal lineages and are not conducive to microglia emergence, hence, to improve the current system,^20^ we generated a co‐culture model of hiPSC‐derived retinal organoid and microglia‐like cells and evaluated its effect on developmental potential.

## 2 MATERIALS AND METHODS

### 2.1 HiPSC culture

HiPSC lines WT1 (SB‐AD2) and WT3 (SB‐AD4)^17^ were cultured in mTeSR1 (Stem Cell Technologies) supplemented with 1% penicillin/streptomycin (Thermo Fisher Scientific) on Matrigel Growth Factor Reduced (Corning)‐coated plates at 37°C and 5% CO_2_ in a humidified incubator. Cell culture medium was replaced daily, and cells were allowed to grow for 4–5 days prior to passaging at a ratio of 1:6 with Versene EDTA (Lonza).

### 2.2 Human microglia culture

Microglial cell line HMC3 (ATCC) was cultured on tissue culture treated plates in EMEM (ATCC) supplemented with 10% FBS and 1% penicillin/streptomycin. Cells were passaged with 0.25% (w/v) Trypsin‐ 0.53 mM EDTA with a subcultivation ratio of 1:3–1:8.

### 2.3 HiPSC differentiation to microglia‐like cells (iMG)

Microglial differentiation was based on a method described by Douvaras et al.^23^ Briefly, WT2 (SB‐AD3) hiPSCs^24^ were plated on Matrigel coated plates at a density of 15,000 cells/cm^2^ in mTeSR1 medium supplemented with 1% penicillin/streptomycin and 10 μM Y‐27632 (Chemdea). After 24 h, media was switched to mTeSR1 and changed daily. Approximately 2–4 days later, when individual colonies became visible, medium was changed to differentiation medium containing mTeSR1 Custom media (Stem Cell Technologies Ref: 85851) supplemented with 80 ng/ml BMP4 (R&D Systems) followed by daily medium changes. On day 4, media was changed to StemPro®‐34 SFM (1×) (with 2 mM GlutaMAX‐I) (Life Technologies) supplemented with 25 ng/ml bFGF (Life technologies), 80 ng/ml VEGF (Gibco) and 100 ng/ml SCF (Peprotech). On day 6, medium was changed to StemPro®‐34 SFM (1×) (with 2 mM GutaMAX‐I) supplemented with 50 ng/ml SCF (Life Technologies), 50 ng/ml IL‐3 (Life Technologies), 5 ng/ml TPO (Life Technologies), 50 ng/ml M‐CSF (Life Technologies), 50 ng/ml Flt3 ligand (Fisher Scientific). On day 10, cells released into the medium were pelleted, resuspended in fresh medium and replated back to the plates. From day 14, the medium was changed to StemPro®‐34 SFM (1×) (with 2 mM GlutaMAX‐I) supplemented with 50 ng/ml M‐CSF, 50 ng/ml Flt3 ligand and 25 ng/ml GM‐CSF and cells were pelleted and replated every 4 days thereafter. For maturation, floating aggregates were dissociated with Tryple Express (ThermoFisher) and replated onto tissue culture treated plates at a density of 40–50 × 10^3^ cells/cm^2^ in RPMI‐1640 with 2 mM GlutaMAX‐I (Life Technologies) supplemented with 10 ng/ml GM‐CSF and 100 ng/ml IL‐34 (Peprotech). Media was changed every 3–4 days. All media was supplemented with 1% penicillin/streptomycin.

### 2.4 HiPSC differentiation to retinal organoids

Retinal organoid differentiation was based on a previously described method.^20^ Briefly, after reaching 80%–90% confluency, hiPSCs were dissociated to form a single cell suspension with Accutase (Thermo Fisher Scientific) and seeded at a density of 7000 cells per well in Lipidure (Amsbio)‐coated 96 well plates in 100 μl mTeSR1 with 10 μM Y‐27632 (designated day −2). On day 0 of differentiation 200 μl of differentiation medium was added, which comprised 41% IMDM, 41% Ham's F12, 15% KOSR, 1% GlutaMAX, 1% penicillin/streptomycin, 1% chemically defined lipid concentrate (all Thermo Fisher Scientific), and 225 μM 1‐Thioglycerol (Sigma), medium was changed every 2 days. At day 6, 2.25 nM BMP4 was added, and medium was changed every 3 days. On day 18, medium was changed to DMEM/F12, 1% GlutaMAX, 1% penicillin/streptomycin, 10% FBS, 1% N2 (all Thermo Fisher Scientific), 0.5 μM retinoic acid (Sigma), 0.1 mM taurine (Sigma) and 0.25 μg/ml Fungizone (Thermo Fisher Scientific) and medium was half‐replenished two to three times a week thereafter. After day 120, retinoic acid was removed from culture medium.

### 2.5 Co‐culture of retinal organoids with iMG

iMG were added at 13 weeks of retinal organoid differentiation according to the following procedure. Half of the culture medium was removed from retinal organoids. After 7–9 days of maturation, iMG were washed with PBS and dissociated with TrypLE Express (1×) for 5–10 min at 37°C. Cells were resuspended in co‐culture medium comprised of retinal organoid medium supplemented with 10 ng/ml GM‐CSF and 100 ng/ml IL‐34 at a concentration of 5000 cells per 100 μl. 100 μl of iMG cell suspension was added to each well with retinal organoids and media was half‐replenished every 3–4 days thereafter.

### 2.6 RT‐qPCR

RNA was isolated using ReliaPrep™ RNA Cell Miniprep System (Promega) following the instructions of the manufacturer. cDNA was synthesized using GoScript™ Reverse Transcription System (Promega) according to the manufacturer's instructions. qPCR was performed in triplicate reactions using GoTaq® qPCR Master Mix reagent system (Promega). The reaction was run on the Applied Biosystems® QuantStudio™ 7 Flex Real‐Time PCR System (Life Technologies). The data were analysed using the QuantStudio™ software (Life Technologies) and relative gene expression was determined using the $2^{-\Delta \Delta C_{t}}$ method using *GAPDH* as a housekeeping gene. The list of oligonucleotides is shown in Table S1.

### 2.7 Flow cytometry

Cells in the supernatant were dissociated into single cell suspension with TrypLE Express Enzyme (1×) (ThermoFisher) and resuspended in 2% FBS in PBS solution with the appropriate concentrations of the antibodies (Table S2) and DAPI (CyStain® DNA, PARTEC). Samples were analysed on BD™ LSR II flow cytometer (BD Biosciences). The data was analysed on BD FACSDiva software (BD Biosciences).

### 2.8 Immunofluorescence

For microglia staining, cells were washed three times with PBS and fixed with 4% paraformaldehyde (PFA) for 10 min. Subsequently, cells were washed three times with PBS and permeabilized with 0.1% Triton‐X100 (Sigma) in PBS for 10 min following by blocking with 10% normal goat serum (Invitrogen) in PBS at room temperature. Primary antibody was diluted in 5% normal goat serum in PBS and samples incubated overnight at 4°C. Cells were washed three times with PBS and incubated with a secondary antibody conjugated to Alexa488 (Life Technologies) diluted in 5% normal goat serum for 1 h at room temperature. Cells were washed three times with PBS and nuclei counterstained with Hoechst 33042 (Life Technologies). Images were acquired with Zeiss Axiovert 200 M – Inverted Widefield microscope with Zeiss AxioCam HRm camera (all Carl Zeiss Microscopy GmbH). For retinal markers, organoids were washed with PBS and fixed in 4% PFA for 20 min followed by three washing steps with PBS. Subsequently, they were transferred to 30% sucrose solution and kept at 4°C overnight followed by embedding in Optimal Cutting Temperature (OCT) embedding matrix (Cell Path Ltd) and cryosectioning. Sections were then rinsed in PBS and incubated for 1 h in blocking buffer (5% normal goat serum and 0.3% Triton‐X‐100 in PBS) at room temperature. All antibodies (Table S2) were diluted in 1% bovine serum albumin (Sigma) and 0.3% Triton‐X‐100 in PBS. Primary antibodies were applied overnight at 4°C. Secondary antibodies conjugated to Alexa488 (Life Technologies) or Cy3 (Jackson Immuno Research Laboratories) were applied for 1 h at room temperature. Sections were washed with PBS and mounted with Vectashield (Vector Laboratories) containing Hoechst 33042 (Life Technologies). Images were acquired with Zeiss AxioImager with Apotome and Zeiss AxioCam camera (all Carl Zeiss Microscopy GmbH).

### 2.9 Phagocytosis

FluoSpheres™ polystyrene, 1.0 μm, orange (540/560) (ThermoFisher Scientific) were added to the cells at a ratio of 200 beads per cell and incubated for 3 h at 37°C. Cells were washed three times with PBS and dissociated with Accutase and resuspended in 2% FBS in PBS solution followed by passing through a 100 μm cell strainer. Nuclei were counterstained with DAPI and DRAQ5 (BioStatus) and extracellular fluorescence quenched with 0.2% Trypan Blue Stain (Life Technologies) for 10 min. Samples were analysed on BD™ LSR II flow cytometer (BD Biosciences). The data was analysed on BD FACSDiva software (BD Biosciences).

### 2.10 Cytokine release

Samples were treated with 100 ng/ml lipopolysaccharide (LPS) for 24 h and assayed for human chemokine and cytokine biomarkers with V‐PLEX Human Cytokine 30‐Plex Kit (Meso Scale Discovery) following the manufacturer's instructions. The analytes included Eotaxin, Eotaxin‐3, GM‐CSF, IFN‐γ, IL‐1α, IL‐1β, IL‐2, IL‐4, IL‐5, IL‐6, IL‐7, IL‐8, IL‐8 (HA), IL‐10, IL‐12/IL‐23p40, IL‐12p70, IL‐13, IL‐15, IL‐16, IL‐17A, IP‐10, MCP‐1, MCP‐4, MDC, MIP‐1α, MIP‐1β, TARC, TNF‐α, TNF‐β, VEGF‐A. GM‐CSF was excluded from the analyses since it was present in the culture medium.

### 2.11 Transmission electron microscopy

Retinal organoids were fixed with 2% glutaraldehyde in 0.1 M sodium cacodylate buffer. Transmission electron microscopy including all cell processing was performed at Newcastle University Electron Microscopy Research Services. Samples were post fixed in 1% osmium tetroxide, dehydrated in gradient acetone, and embedded in epoxy resin. Ultrathin 70 nm sections were picked up on copper grids, stained with uranyl acetate and lead citrate and imaged using a Philips CM100 transmission electron microscope with high‐resolution digital image capture.

### 2.12 Single cell (sc) RNA‐Seq

Retinal organoids were dissociated to single cells using the Neurosphere Dissociation kit (P) (Miltenyi Biotech) according to the manufacturer's protocol. Cell capture and library generation was carried out using the Chromium Single Cell 3′ Library & Gel Bead Kit, version 3 (10× Genomics). scRNA‐Seq libraries were sequenced to 50,000 reads per cell on an Illumina NovaSeq 6000. The sequencing data was demultiplexed, aligned to the human reference genome GRCh38 using CellRanger version 3.01. A quality control step was used to remove any cells where fewer than 1000 reads or 500 genes or >15% mitochondrial reads were detected. Doublets were removed using DoubletFinder (version 2.0.3.). Seurat (version 3.1.3) was used to analyse the data. The samples were log normalized. 2000 highly variable genes were selected and a PCA component analysis was performed. The Seurat integration workflow was performed to remove batch effects between the samples. The first 30 principal components were chosen for sample integration. Clustering analysis was then performed on the combined dataset using a resolution of 0.5. Uniform manifold approximation and projection (UMAP) was used to visualize the clusters. The Wilcoxon test was used to identify marker genes for each cluster.

### 2.13 Electrophysiological recordings

Electrophysiological recordings were performed as described in Dorgau et al.^25^ Briefly, 24 h prior to recordings retinal organoids were incubated with 9‐cis‐retinal (10 nM; Sigma‐Aldrich). After several washes in artificial cerebrospinal fluid (in mM: 118 NaCl, 25 NaHCO_3_, 1 NaH_2_PO_4_, 3 KCl, 1 MgCl_2_, 2 CaCl_2_, 10 glucose, 0.5 l‐Glutamine and 0.01 9‐cis‐retinal; 34°C) to remove culture medium, retinal organoids (7–8 per condition) were opened longitudinally and placed with the presumed retinal ganglion cell (RGC) layer facing down onto a 4096 channel multielectrode array (MEA). The organoids settled down for at least 2 h before recordings which were performed on the BioCam4096 MEA platform with HD‐MEA Arena chips (3Brain GmbH). Full field white light pulses (WLPs, 200 ms, 217 μW/cm^2^ irradiance, 1 Hz) were flashed for 5 min onto the organoids following recording spontaneous activity in the dark for 5 min. Extraction of spikes from raw traces was done using a quantile‐based event detection^26^ and single unit spikes were sorted by an automated spike sorting method for dense, large‐scale recordings.^27^ Firing rate analyses and statistical significance (Mann–Whitney test) were evaluated using Matlab (Mathworks) and Prism (GraphPad). Retinal ganglion cells were considered responsive if they changed their spiking activity by at least 25% (increase or decrease) during 30 s after WLP onset compared to the similar time window before the light stimulus (dark condition). The mean % change (±SEM) in activity between windows was calculated and plotted using Prism (GraphPad).

## 3 RESULTS

### 3.1 Differentiation and functional characterization of hiPSC‐derived microglia‐like cells

**FIGURE 1:**
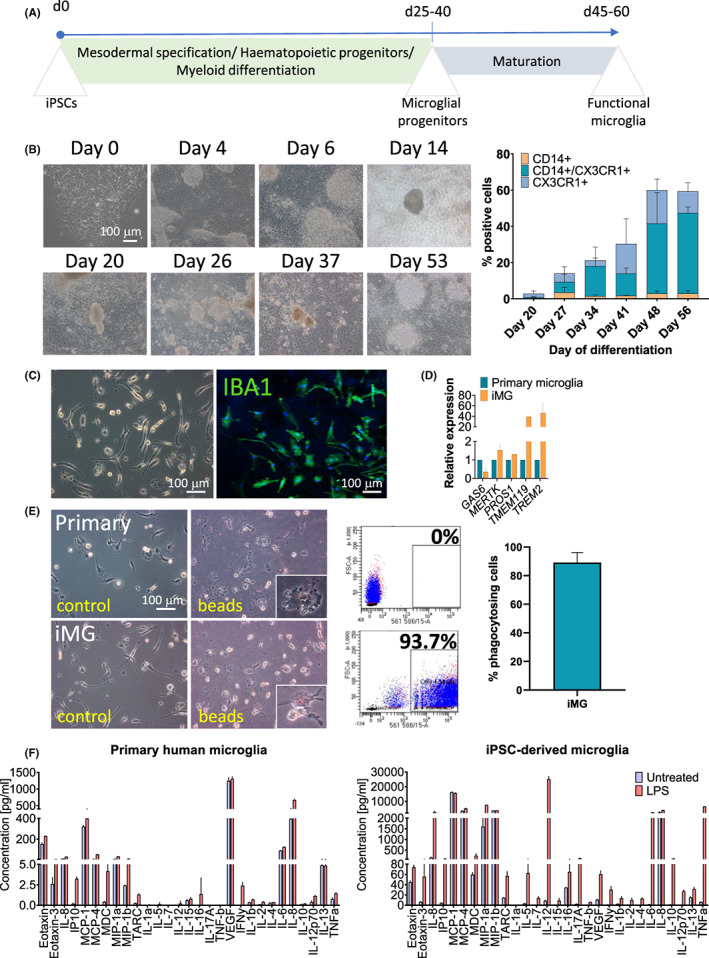
Differentiation and functional characterization of iMG cells. (A) Schematic representation of the differentiation protocol to microglia‐like cells. (B) Morphological changes observed throughout the differentiation. Flow cytometric analysis showing increased levels of CD14 and CX3CR1 positive cells over time (mean ± SEM, *n* = 3). (C) Bright‐field images showing cells displaying ramified morphology with immunofluorescence analysis confirming cells being positive or IBA1. (D) Gene expression analysis comparing iMG to a control cell line confirming comparable or increased levels of gene expression (mean ± SEM, *n* = 2). (E) Changes in cell morphology after the addition of fluorescent beads showing cells acquiring amoeboid morphology. Flow cytometry analysis showed that ~90% of cells were phagocytosing. Insets show cells with and without the addition of the fluorescent beads (mean ± SEM, *n* = 3). (F) Cytokine release 24‐h after LPS stimulation showing comparable or increased levels of cytokine release by iMG comparing to primary microglial cell line (mean ± SEM, *n* = 2). iMG, hiPSC‐derived microglia‐like cells; LPS, lipopolysaccharide.

Microglia‐like cells (iMG) were generated from hiPSCs by first deriving cells of mesodermal lineage following myeloid differentiation towards microglial progenitors (Figure 1A).^23^ Over time, there was an increase in the number of budding myeloid progenitors released into the supernatant. Flow cytometric analysis showed that there was a steady increase in the number of microglial progenitor cells positive for CD14 and CX3CR1 over time (Figure 1B). When the percentage of CD14 positive cells was over 40%,^23^ cells were dissociated and replated as adherent cultures for maturation. Over time cells started to display ramified morphology and were positive for the microglial marker IBA1 (Figure 1C). Although microglial transcriptomic signature can be region‐specific and dynamic, a unique microglial signature was defined by Butovsky et al.^28^ We compared our iMG cells to a primary microglial cell line and found that the cells had comparable or higher expression levels of TAM (TYRO3, AXL and MERTK)‐related genes *MERTK, PROS1* and *GAS6*, as well as others including *TMEM119* and *TREM2* (Figure 1D). We performed functional characterization of iMG cells and found that they were able to phagocytose fluorescent beads as illustrated in Figure 1E, where after exposure to the beads, cells changed their morphology from semi‐activated state in vitro to amoeboid suggesting adaptation of an activated state. Quantification analyses by flow cytometry showed that ~90% of cells had phagocytic capacity. In addition, we used endotoxin challenge to assess the cytokines released upon stimulation with LPS over 24 h. Twenty‐nine targets were assayed and iMG robustly responded to LPS and upregulated all cytokines tested apart from IL‐1α, and to a higher level than primary microglia (Figure 1F).

### 3.2 Generation of retinal organoids containing iMG cells

**FIGURE 2:**
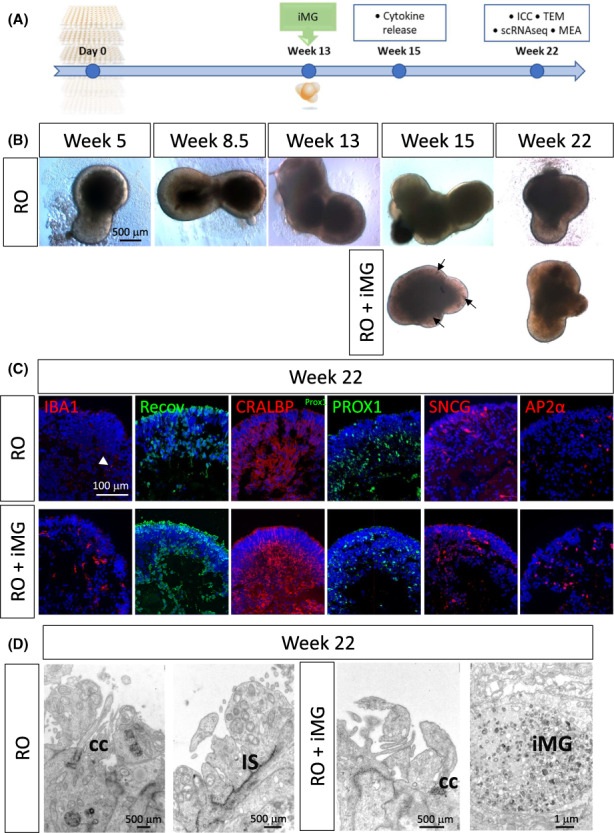
Characterization of retinal organoid and iMG co‐cultures. (A) Schematic representation of the timeline for initiating the co‐culture experiments and time points for various assays. (B) Bright‐field images of retinal organoids over the course of the differentiation showing no clear differences in morphology with and without the incorporation of iMG. Putative iMG incorporated into the organoid are shown with arrows in week 15 organoids. By week 22, iMG are incorporated into the organoid and are rather difficult to detect with BF images. (C) Immunofluorescence images showing presence of key retinal cell types in retinal organoids and the co‐cultures, including photoreceptors (Recov), Muller Glia (CRALBP), differentiating neurons of the inner nuclear layer (PROX1), retinal ganglion cells (SNCG), amacrine cells (AP2α), and microglia (IBA1) – only present in the co‐cultures. White arrow indicates background signal (*n* = 8–10). (D) Ultrastructural characterization of retinal organoids with and without iMG showing presence of connecting cilia, inner segments, and microglia‐like cells in the co‐cultures. CC, connecting cilia; IS, inner segment; ICC, immunocytochemistry; iMG, hiPSC‐derived microglia‐like cells; MEA, multielectrode array; scRNA‐Seq, single cell RNA sequencing; RO, retinal organoid; TEM, transmission electron microscopy

Two control hiPSC lines were differentiated to retinal organoids using an established protocol.^20^ Since microglial progenitors arise from mesodermal lineage, unlike the retina which develops from neuroectoderm, not allowing for microglial development in situ, we added iMG to retinal organoids after 13 weeks in culture (Figure 2A), which is in line with the developmental timeline of microglial migration to the retina, reported to take place between post‐conception week (PCW) 6 and PCW17.^2^, ^3^, ^4^ We monitored the development of the organoids over time until week 22. Organoids developed a phase‐bright apical layer of neuroepithelium and at weeks 15 and 16 iMG cells could be detected on the surface and within the organoids (Figure 2B; Figure S1). The organoids also displayed the presence of photoreceptors (Recoverin), Muller glia (CRALBP), differentiating neurons of the inner nuclear layer (PROX1), retinal ganglion cells (Gamma‐synuclein; SNCG) and amacrine cells (AP2α). IBA1 positive cells were detected in the co‐culture organoids at 22 weeks (Figure 2C). Ultrastructural morphology assessment with transmission electron microscopy indicated that organoids with and without iMG developed connecting cilia, inner segment, and in some cases primitive outer segments. We were also able to detect presumptive iMG cells, which contained lipidic inclusions and resembled a morphology of a stimulated macrophage^29^ (Figure 2D).

### 3.3 Transcriptomic analysis of iMG‐retinal organoid co‐cultures

**FIGURE 3:**
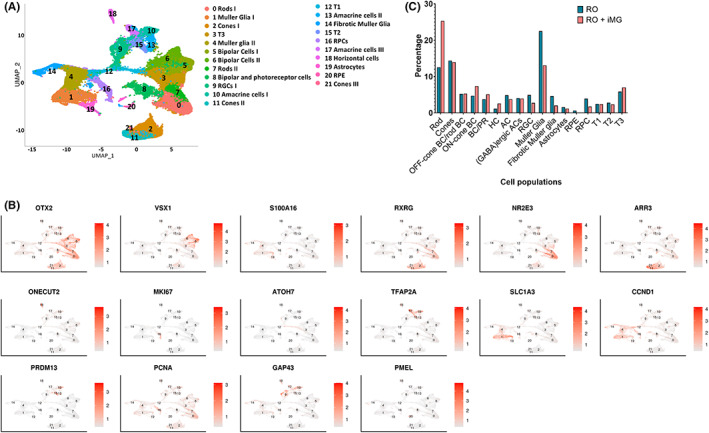
Single cell RNA‐Seq of retinal organoid and iMG co‐cultures. (A) UMAP showing the cell clusters in retinal organoids in the presence and absence of iMG, revealing the presence of 21 clusters. (B) Expression of key retinal cell type markers genes used to facilitate cluster definition overlaid onto the UMAP. (C) Graph showing the frequency of all cell types in the retinal organoids generated in the presence and absence of iMG (*n* = 1 experiment, 12 ROs per condition). AC, amacrine cell; BC, bipolar cell; HC, horizontal cell; iMG, hiPSC‐derived microglia‐like cells; PR, photoreceptor; RGC, retinal ganglion cell; RO‐retinal organoids; RPC, retinal precursor cell; RPE, retinal pigment epithelium

To assess the development of retinal organoids in the absence and presence of iMG, scRNA‐Seq was carried out at 22 weeks of differentiation. Following quality control, ~51,000 cells were obtained and merged using the Seurat package. Transcriptionally similar cells were grouped together and visualized using UMAP, which revealed the presence of 21 cell clusters (Figure 3A). The highly and differentially expressed genes (Table S3) were used to define cluster identity (representative examples shown in Figure 3B). All the key retinal cell types were identified including photoreceptors, interneurons, RGCs, Muller glia as well as transitional cell populations recently reported by Sridhar et al.^21^ alongside astrocytes, RPE and fibrotic Muller glia cells. To our surprise, we did not detect a cluster with gene markers of microglial/ macrophage identity, despite the fact that we detected a small number of IBA1 positive cells by immunofluorescence and presumptive microglial‐like cells by TEM (Figure 2C,D). We hypothesized that only a small number of iMG remained within the organoids after 8.5 weeks of co‐culture, and these remaining cells could have been lost during the single cell dissociation in preparation for scRNA‐Seq. Future work will include optimizing the final medium composition to allow long‐term co‐culture and survival of a larger number of iMG cells.

Since the iMG were added at 13 weeks of differentiation, we assessed the impact of their incorporation into retinal organoids at 22 weeks by comparing the percentage of various retinal cell clusters in control and iMG‐retinal organoids (Figure 3C). This analysis revealed similar cell type frequency except for Rods, occurrence of which was higher in iMG‐retinal organoids. Muller glia cell presence was also noticeably lower. The interaction between microglia and Muller glia is well documented in the literature,^30^ thus further work is needed to better understand the impact of iMG on changes in the rod and Muller glia cell occurrence within the retinal organoids.

### 3.4 Functional characterization of retinal organoids containing iMG

**FIGURE 4:**
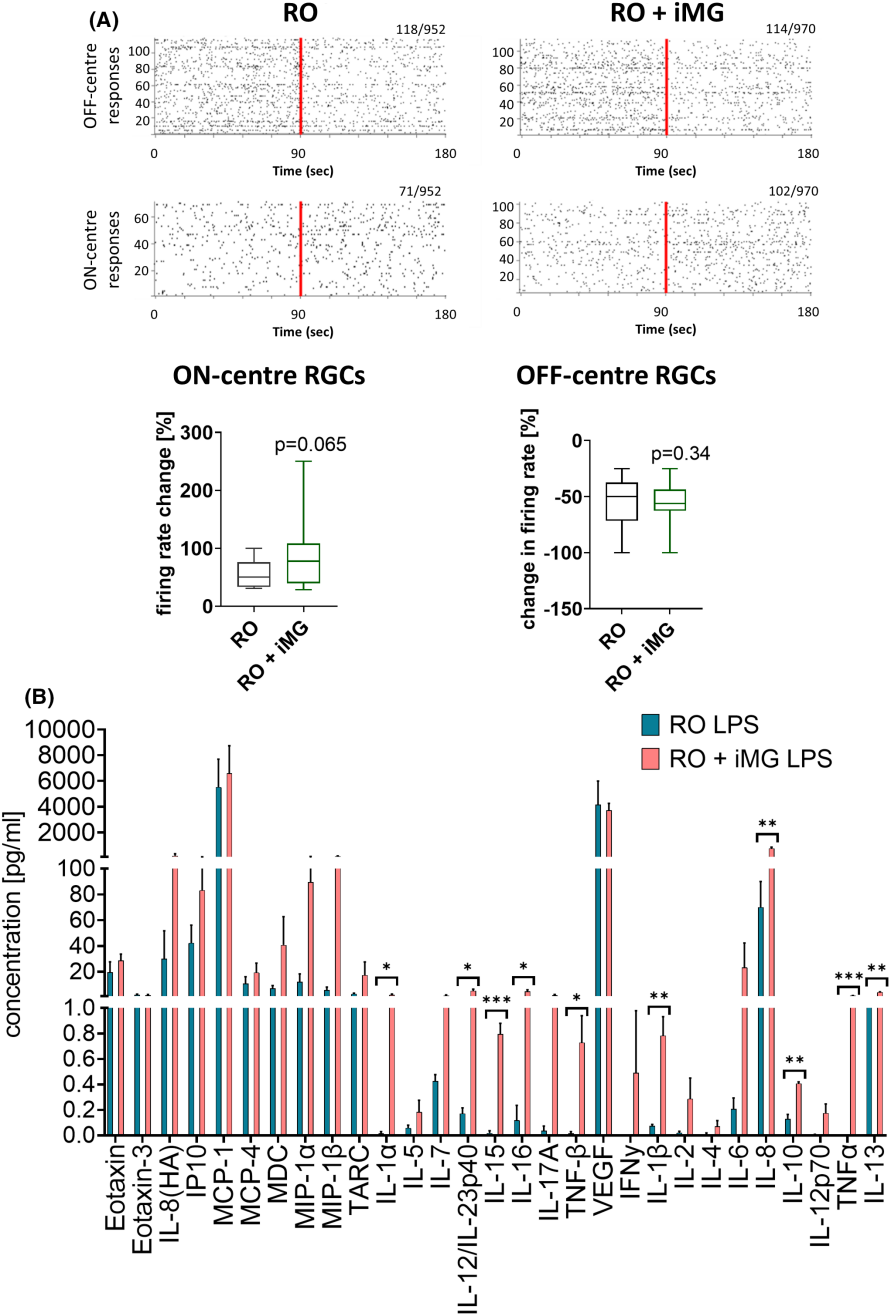
Functional characterization of retinal organoids and iMG co‐cultures. (A) Spike raster plots from putative Off‐centre retinal ganglion cells (RGCs, top) and ON‐centre RGCs (bottom) of retinal organoids with (RO + iMG) and without microglia (RO) revealed either a decreasing firing rate for Off‐centre RGCs or an increasing spiking activity for ON‐centre RGCs after WLPs. Each row in raster plots represent a different RGC and each vertical bar represents a spike from the corresponding RGC. Numbers of responding RGCs and the total amount of recorded cells are stated on the top right corner of each spike raster plot. The red line illustrates the pulsed stimulus onset whereas the left half before indicates the spontaneous activity before WLP exposure and the right half when exposed to WLP. Box plots indicate no statistical differences in percentage (%) firing rate change of putative ON RGC (left) and OFF RGCs (right) after WPL in RO and RO + iMG condition (Mann–Whitney test; *p* = 0.065 for ON RGCs and *p* = 0.34 for OFF RGCs). The box plot shows the median and interquartile ranges with Tukey whiskers. *n* = 1 experiment, 7–8 retinal organoids per condition. (B) Cytokine release measurements showed increased levels of cytokines released in the media after exposure to LPS in retinal organoids containing iMG comparing to organoids alone (mean ± SEM; *n* = 3; unpaired *t*‐test). iMG, hiPSC‐derived microglia‐like cells; LPS, lipopolysaccharide; RGC, retinal ganglion cell; RO, retinal organoid

The impact of incorporating iMG on physiological function of retinal organoids was assessed using multielectrode ganglion cell recordings. Retinal organoids with and without iMG showed a decrease in their spiking activity after WLPs (Figure 4A), indicating putative OFF centre‐like responses from RGCs without any significant differences either in number of responding RGCs or their mean spiking activity change (11.8% in RO + iMG and 12.4% in RO) (Figure 4A, *p* = 0.34). Similarly, putative ON centre‐like responses of RGCs were found in both conditions, revealing a firing rate increase after WLP and showing a statistical trend in % firing rate change in the presence of iMG, with a slight increase in the number of responding RGCs in the retinal organoids with iMG (10.5% in RO + iMG and 7.5% in RO) (Figure 4A, *p* = 0.065).

Next, we asked whether LPS elicited an immune response in situ by challenging the organoids at 15 weeks for 24 h and used the same panel of human cytokines as we did for iMG cultured on their own (Figure 1). After 24‐h treatment, we saw an upregulation in the levels of most cytokines, including levels of ILα, IL‐12/IL‐23p40, IL‐15, IL‐16, TNF‐β, IL‐1β, IL‐8, IL‐10, TNF‐α and IL‐13 being significantly higher in organoids containing iMG (Figure 4B).

Overall, the cytokine response to LPS was lower than the results obtained from isolated cultures. This is in line with published literature on cerebral organoids containing microglia and can be explained by iMG representing a low proportion of cells comparing to other cell types within the organoid.^22^

## 4 DISCUSSION

Increasingly, it is recognized that there is a need for more physiologically relevant in vitro systems including an immune component. Several studies described invasion of cerebral organoids with hiPSC‐derived microglial cells,^31^, ^32^, ^33^ including a report that demonstrated utility of the co‐culture system in the study of Alzheimer's disease.^34^ However, to our knowledge there are no current immunocompetent models of retina derived from hiPSCs. Retinal organoids are increasingly being used for a variety of applications, including the study of basic principles of development,^35^, ^36^ disease modelling,^17^, ^19^, ^37^ development of novel therapeutic strategies,^38^, ^39^ regenerative medicine^40^, ^41^ and drug safety assessment.^20^, ^42^ Their use in the pre‐clinical setting could be extended and enhanced by increasing several characteristics that mimic the retina in vivo. Current models lack some of the key features, including vascularization, endothelial cells and immune cells, such as microglia.

In this study we aimed to address one of these limitations by incorporating microglial‐like cells derived from hiPSCs into retinal organoids. Here, we show that functional human microglia‐like cells can be derived from hiPSCs and integrated into retinal organoids. The key findings were that microglia‐like cells were able to integrate into the organoids and were able to respond to endotoxin challenge demonstrating retention of at least some of their functionalities. Our data showed a significant upregulation of several pro‐inflammatory markers in response to endotoxin challenge, including ILα, IL‐12/IL‐23p40, IL‐15, IL‐16, TNF‐β, IL‐1β, IL‐8 and TNF‐α. Interestingly, we also observed increased levels of an anti‐inflammatory cytokine IL‐10, which is known to have a neuroprotective function and prevent inflammation‐mediated neurodegeneration, and reduce retinal microglial migration in response to LPS.^43^ In addition, we also saw an upregulation in another anti‐inflammatory mediator IL‐13, which has been previously shown to reduce ocular inflammation in response to LPS^44^ and as a modulator of inflammation associated with uveitis.^45^ This demonstrates that our system is able to replicate the balance between pro‐ and anti‐inflammatory conditions. Using hiPSC‐derived microglial‐like cells as opposed to primary cells has several advantages, the major ones being the ability to generate cells at scale and reduce the effect of donor variability. Future work would require further investigating optimal co‐culture conditions to allow long‐term survival of iMG, including further co‐culture medium optimization and potentially the addition of using a microfluidics platform. Regional heterogeneity of microglial gene signatures is one of their most distinctive features. Future work should also address the possibility of generating brain/retina specific iMG via genetic and epigenetic tools.

There are multiple applications where immunocompetent model of human retina would be of value. There are increasing reports of intraocular inflammation following adeno‐associated virus (AAV) therapy, which leads to the reduction of therapeutic efficacy. Although the pathways involved are complex, retinal microglia is one of the components of the innate immune response to AAV‐mediated retinal gene therapy.^46^ Studying the immune cells in a relevant microenvironment to human can provide advantages to using animal models which have physiological and functional differences compared to human retina. Furthermore, having an in vitro alternative would reduce the number of animals used in pre‐clinical research. Lastly, inclusion of microglia in in vitro human retinal models would allow mechanistic evaluations and finding new therapies for several diseases that have been linked to microglial dysfunction, including age‐related macular degeneration,^47^ autoimmune responses in the retina^48^ and diabetic retinopathy.^49^

In conclusion, we demonstrated the feasibility of generating an immunocompetent in vitro model of functional retina derived from hiPSCs. Incorporation of microglia‐like cells did not prevent normal retinal organoid development and establishment of functionality. Future work would require testing the ability of the model to mimic disease phenotypes with a known microglial component. In addition, other areas of research such as finding new gene therapies and assessing drug safety would benefit from this model.

## AUTHOR CONTRIBUTIONS

**Valeria Chichagova:** Conceptualization (equal); data curation (equal); formal analysis (equal); investigation (equal); methodology (equal); supervision (equal); validation (equal); visualization (equal); writing – original draft (lead). **Maria Georgiou:** Data curation (supporting). **Madeleine Carter:** Data curation (supporting). **Birthe Dorgau:** Data curation (equal); formal analysis (equal); writing – review and editing (supporting). **Gerrit Hilgen:** Data curation (equal); formal analysis (equal); writing – review and editing (supporting). **Joseph Collin:** Data curation (equal); writing – review and editing (supporting). **Rachel Queen:** Formal analysis (equal). **Git Chung:** Data curation (equal). **Jila Ajeian:** Data curation (supporting). **Marina Moya‐Molina:** Data curation (supporting). **Stefan Kustermann:** Writing – review and editing (equal). **Francois Pognan:** Writing – review and editing (equal). **Philip Hewitt:** Data curation (supporting); writing – review and editing (equal). **Michael Schmitt:** Data curation (supporting); writing – review and editing (equal). **Evelyne Sernagor:** Conceptualization (equal); formal analysis (equal); funding acquisition (equal); writing – review and editing (equal). **Lyle Armstrong:** Conceptualization (equal); funding acquisition (equal). **Majlinda Lako:** Conceptualization (equal); data curation (equal); formal analysis (equal); funding acquisition (equal); investigation (equal); methodology (equal); project administration (lead); resources (lead); supervision (lead); validation (equal); visualization (equal); writing – original draft (lead).

## CONFLICT OF INTEREST

The authors confirm that there is no conflict of interest.

## Supporting information


Figure S1

Table S1
Table S2

Table S3

## Data Availability

scRNA‐Seq data files were uploaded to the Gene Expression Omnibus (GEO) database (submission GSE173180).
