# Supplementary figures and images for: Incorporating microglia‐like cells in human induced pluripotent stem cell‐derived retinal organoids
Source: J Cell Mol Med. 2023 Jan 16;27(3):435–45. doi: 10.1111/jcmm.17670 (PMC9889627; doi:10.1111/jcmm.17670)

Figure S1.

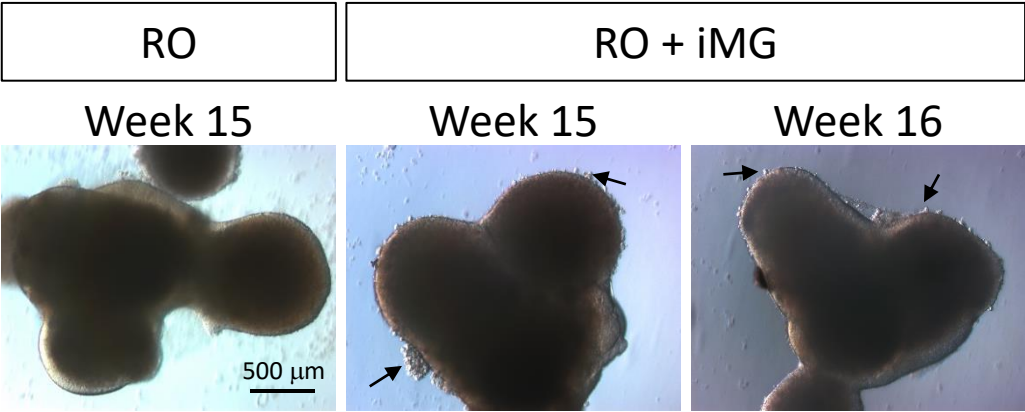

Supplement: Supplementary file 1 — Figure S1 [file JCMM-27-435-s001.pdf]
