# Supplementary material for: Incorporating microglia‐like cells in human induced pluripotent stem cell‐derived retinal organoids
Source: J Cell Mol Med. 2023 Jan 16;27(3):435–45. doi: 10.1111/jcmm.17670 (PMC9889627; doi:10.1111/jcmm.17670)
Supplement: Supplementary file 2 — Table S1 Table S2 [file JCMM-27-435-s002.docx]

**Supplementary Table 1. List of oligonucleotides.**

| **Gene** | **Forward primer** | **Reverse primer** |
| --- | --- | --- |
| *GAPDH* | TGCACCACCAACTGCTTAGC | GGCATGGACTGTGGTCATGAG |
| *GAS6* | ACGACCCCGAGACGGATTAT | GGCGAAGCCTGAGTTTTTGG |
| *MERTK* | AGCCTGAGAGCATGAATGTCACCA | TGTTGATCTGCACTCCCTTGGACA |
| *PROS1* | AGAGGCAAACTTTTTGTCAAAGCA | AGACCTCCCTGGCTTCTTCT |
| *TMEM119* | AGCACGGACTCTCTCTTCCAG | GTGCCCCCAGGACCAGTTC |
| *TREM2* | CCCACCCACTTCCATCCTTC | CTCAGCCCTGGCAGAGTTTG |

**Supplementary Table 2. List of antibodies.**

| **Antibody** | **Host** | **Dilution** | **Supplier** |
| --- | --- | --- | --- |
| RECOVERIN | Rabbit | 1:1000 | Millipore, ab5585 |
| AP-2α | Mouse | 1:100 | Santa Cruz Biotechnology Inc., sc-12726 |
| Synuclein gamma (SNCG) | Mouse | 1:500 | Abnova, H00006623-M01A |
| CRALBP | Mouse | 1:100 | GeneTex, GTX15051 |
| PROX1 | Rabbit | 1:1000 | Millipore, ab5475 |
| IBA1 | Rabbit | 1:400 | Wako, Ref:019-19741 |
| CX3CR1 PE-conjugated | Mouse | N/A | R&D systems, FAB5204P-025 |
| CD14 APC-conjugated | Mouse | N/A | Bio-Rad, MCA596APCT |
